# A Systematic Review Evaluating the Use of Mixed Reality Technologies Within Undergraduate Medical Anatomical Teaching

**DOI:** 10.7759/cureus.110021

**Published:** 2026-06-01

**Authors:** Jenny Saleema N Kakonge

**Affiliations:** 1 General Internal Medicine, University of Southampton, London, GBR

**Keywords:** anatomy teaching and learning, augmented reality (ar), computer-based learning, undergraduate and graduate medical education, virtual reality (vr)

## Abstract

Technological advances have shifted undergraduate medical education toward computer-assisted learning (CBL). The recent increase in CBL has most noticeably influenced anatomical curricula.

Virtual reality (VR) and augmented reality (AR) are emerging computer technologies that modify reality and stimulate senses through contrasting methods. VR submerges individuals within a virtually constructed pseudo-reality using specialist headsets (head-mounted devices). AR merges digitalised images into our natural visual fields, altering reality without a complete digitalised environment. VR and AR can be collectively referred to as mixed reality technologies (MRT). Both technologies are extensions of gaming technologies, which have been increasingly modified to fit into non-gaming applications through a process of ‘gamification.’

Technological progression and increased commercial accessibility of MRT have furthered the interest in incorporating these technologies within medical education, with an interest in gross anatomical teaching, as visualisation is a compound element of this area of medical science. With technology becoming a great focus within education, there is potential for a paradigm shift, changing how anatomical teaching is delivered. A systematic review was conducted to assess current applications of MRT in undergraduate gross anatomy teaching and whether the use of MRT within gross anatomical teaching influences academic performance.

Four databases, MEDLINE, Scopus, Web of Science and ERIC, were searched for educational scientific papers. A total of 608 papers were selected to undergo title and abstract screening, out of which seven eligible papers were identified for full analysis and review.

The outcome of this review was that VR and AR are as effective as traditional teaching modalities. Three out of the seven studies reported statistically significantly higher academic scores with MRT use, whilst the rest reported no statistically significant difference between the use of MRTs and traditional modalities.

There is strong evidence for the use of MRTs as supplementary teaching modalities in undergraduate gross anatomical teaching.

## Introduction and background

Pedagogical practises in medical education

Pedagogical practises heavily inform how medical education is delivered. Conventionally, the didactic theory of learning has been applied in medical education. Didactic learning is focused on conveying information in a descending hierarchical format, directly from teacher to student [1]. It is a teacher-centred practise, and students are only passively involved in their own learning experiences. Didactic teaching in medical education can be exemplified by lectures and instructional tutorials, which form a large basis of how medical education has been traditionally taught [1]. 

This principle of teaching has historically been very beneficial in medical education, considering the depth of knowledge and large quantity of information that medical students need to learn and conceptualise, in readiness for clinical practice. 

However, this approach to teaching has become increasingly contested in favour of pedagogical practises which encourage greater student involvement and independence in learning [2].

The pedagogy of medical education has undergone a drastic change and is now increasingly based on the theories of guided discovery learning and interactive learning [3]. Guided discovery learning permits active student participation in knowledge discovery [3,4], as depth of learning is directly reflective of the degree of active involvement. With guided discovery learning, the aim is to facilitate deeper learning of medical knowledge through problem solving [3,4]. Problem-based learning (PBL) is a derivative of this theory [4,5] and is now also a core feature of many medical curricula [5]. PBL enlists the use of higher intellectual skills (problem solving, critical thinking) [6], which, as mentioned, facilitates deeper learning and knowledge consolidation, but also reflects essential qualities of competent doctors [7]. 

The interactive learning theory is based on the epistemological theory of constructivism [6,7], which centres students at the core of their teaching initiative. Like guided discovery learning, this also achieves attainment of higher-order skills, as per the Bloom’s taxonomy framework [8]. In practice, interactive learning involves the use of a digital interface that enables reciprocal interactions between students and teachers [9]. 

The insurgence of technology in medical education has allowed for the increased practise of interactive teaching [9]. This is mostly exemplified by the prevalence of computer-based learning (CBL), which is a derivative of interactive learning [9].

Overall, continuous adaptations and advancements in our healthcare systems, caused by changing health population demographics, are the main propellant for the transitional changes occurring within medical education. The conclusive aim of this is to provide greater clinical context to undergraduate medical education and to better prepare ‘tomorrow’s doctors’ [10,11].

Anatomical sciences in medical education

Anatomy has been centred as the foundation of medical education [12]. It can be said that anatomy informs all other medical sciences. On a gross scale, anatomy can be defined as the study and analysis of biological structures and human morphology, at both microscopic and macroscopic levels [13]. 

The core purpose of anatomical teaching is to provide transferability to clinical practise [12,13]. Anatomy provides the basis that informs clinical logic and inference, thus permitting clinical problem-solving necessary to combat disease and morbidity.

Anatomy also integrates the recognition of normal physiological function with normal biological structure [14]. This is achieved by introducing the principle of spatial orientation, which enhances students’ conceptualisation of the human body [14].

Pedagogical practices in anatomical education

Traditionally, anatomical sciences have been taught through didactic means of teaching, notably lecture series supported by instructive dissection and prosection [15]. This style of teaching has been based on the use of cadaveric specimens. Using cadavers provides a substantial foundation for the conceptualisation of physiological and pathological processes. Thus, the knowledge attained from cadaver-based anatomy teaching has also proven to be highly clinically transferable [16], and hence, that is why, this is held as the ‘gold standard’ [17] of anatomy teaching. However, there are notable limitations with this, which presently incentivise the development of alternative means of teaching anatomy to medical students. 

A key limitation is the limited availability of cadaveric specimens, alongside the significant costs of maintaining anatomy laboratories, which necessitate specialist facilities, ongoing upkeep and skilled anatomists to support effective teaching and specimen preservation [15-17].

Furthermore, the decline in cadaveric specimens available for dissection has steadily reduced dissection teaching at many medical schools [17]. This has created apprehensions concerning the long-term availability and plausibility of dissection teaching in anatomy. As prosection also utilises cadavers, the future sustainability of this is also encompassed within these apprehensions.

Current research [18] has shown a decline in allocated time for anatomy sessions in medical curricula. This is a reflection of the increased focus on non-clinical ‘soft skills’ [19], such as communication and teamwork, which are nonetheless as essential [11] for future doctors. This decline has challenged educators and facilitators with the objective of retaining the standard of anatomical teaching but altering the means of delivery, in order to adapt to these exogenous changes.

In anatomical teaching, the shift toward increased employment of guided discovery learning and higher student active engagement has been complemented by CBL [20].

Virtual reality (VR) and augmented reality (AR), which collectively are known as mixed reality technologies (MRTs), are paradigms of CBL [21,22]. Both of these technologies are becoming progressively more prevalent in anatomy teaching [23]. 

Mixed reality technology in gross anatomical teaching

MRT technology has been acknowledged to be well adapted for organ visualisation, landmark identification and promoting learning and coherence [24]. In the context of medical anatomy, MRTs have mostly been used as supplementary teaching modalities [25].

Three-dimensional technology, specifically VR and AR, provides a viable alternative which incorporate technology whilst allowing retention of high structural fidelity. This is essential in order to provide the clinical context and transferability necessary to fulfil the objective of preserving anatomical knowledge as a clinician. 

Virtual reality

VR is an immersive computer technology that generates a stimulated pseudo-environment that enables haptic-feedback, sensory and auditory stimulation [25-27]. A head-mounted display (HMD) is used to fully encapsulate the individual within the virtual environment. A digital interface, which can be manipulated, is then generated by computer software and projected onto the display compartment of the HMD [25].

It provides an immersive and interactive approach to anatomical education, enabling detailed visualisation of complex anatomical structures, which would otherwise be difficult to appreciate. This enhances spatial awareness, supports conceptual understanding and facilitates learning of both gross and microscopic anatomy [25].

The use of an HMD is pivotal to the concept of VR [25]. VR allows for real-time tracking of rotational head movements through the HMD. This tracking is also coupled with the tracking of eye movements, which, when combined, permits point-of-view visuals on the display compartment of the HMD, creating the illusion of being immersed within the computer stimulation [25]. This also provides the basis for the stereoscopic graphics, illustrated through VR, which add to the sense of realism, achieved when using VR [26]. In the context of medical anatomy, this would be beneficial for point-of-view dissection, which would replicate the actual process of dissecting, but on a virtual cadaver. As well as this, the tracked head and eye movements in coordination with the display would permit close and multi-angled inspection of virtual three-dimensional organs, muscles, bones and nerves. 

Furthermore, previous VR technologies had reportedly less advanced HMDs, which were associated with unpleasant side effects, that acted as a deterrent for the utilisation of VR [26] in general. These effects were due to poor technological developments, which inaccurately coupled eye and head movement to that of the computer graphics on the display, resulting in headaches and motion sickness in some instances. 

As well as this, the overall accessibility of VR was limited due to the high costs associated with the necessary technology, namely, the HMDs, but also the associated computer software [27]. 

However, current market versions are readily available and optimised for consumer markets with Google (Daydream View) (Google LLC, Mountain View, CA), Samsung (Gear VR) (Samsung Electronics Co., Ltd., Suwon, South Korea) and Oculus (Oculus Rift S) (Meta Platforms, Inc., Menlo Park, CA) having their own HMDs or ‘VR headsets’ on the market. Naturally, this has in turn reduced the overall cost of HMDs, allowing for VR to become increasingly more accessible.

With this in mind, the potential for the application of VR on a broad scale, such as undergraduate medical education, specifically gross anatomy teaching, is less improbable. 

Generally, within medicine, VR has principally been used to supplement post-graduate specialist training with a distinctive focus on surgery [28] through enhancement of visualisation and surgical procedures. Yet newly developed digital applications are enabling adjunct VR and AR technology to be applied in undergraduate medical teaching, as it has been in post-graduate training.

The Anatomage table [29] is a virtual dissection application that exemplifies this emergence of new innovation in anatomy education. It is a large interactive tablet application which employs the use of three-dimensional VR technology amalgamated with reconstructed digital images of real cadaveric specimens [30-32], creating an alternative to fresh cadaveric dissection or prosection. As of now, the software application offers six different ‘virtual cadavers’ and is optimised for dental and medical anatomical teaching as well as teaching of physiology in relation to anatomy [29]. As new as this may seem, the Anatomage table has already been introduced to several British medical institutions [29].

Augmented reality

AR is contrasting to VR, as it does not necessitate complete occlusion through a digital interface. Instead, AR technology projects virtual images onto the real world through a computer or mobile device [33], enhancing the perception of reality, without complete distortion. It is an interactive technology that uses multimedia materials and permits real-time feedback.

AR technology is facilitated through two software applications, which have distinct implementation styles. Marker-based AR relies on recognised markers, which can be two-dimensional or three-dimensional. Instances of these include human faces, innate objects or coded barcodes (quick response (QR) codes) [34]. The recognition of these markers triggers the illustration of the augmented visual graphics. Unmarked AR, however, utilises infrared technology and geographical location data to configure a three-dimensional visual animation, which is often confined to a particular location.

Due to its relative ease of access and readily availability of its supporting technology (mobile technology) [34,35], AR technology is already widely utilised within medical education as an extension of CBL, through tablets and mobile applications [34]. Again, this has currently been in the context of a supplementary learning tool. 

The Human Anatomy Atlas (‘visible human’) [35] is a notable example of an AR system. The advantage of this software application is that it relays the participant’s own physical motions with those of the stimulated digital interface, creating an interactive learning environment, which potentiates a greater depth of learning [36].

The magic mirror (‘miracle’) [37] is another AR system adapted for anatomical teaching. This application acts as a mirror by scanning participants and reflecting this image onto its screen, making it akin to mirror reflections. 

Real medical images (CT scans) are then manipulated onto the reflection. This augmented image can then be further manipulated and tailored to the students’ learning.

Current research

Presently, there is a limitation in research that investigates the efficacy of MRT use and its long-term effectiveness. However, through the limited current literature, the following benefits have been attributed to the use of VR and AR in teaching anatomy.

Research has shown high student satisfaction and motivation, in relation to the use of MRTs in undergraduate medicine anatomy teaching [37,38]. This is an imperative factor to consider, as studies have shown a causative link between high academic competency and student motivation and productivity [39]. 

Firstly, using MRT is supportive of guided discovery learning, as it does not require an instructive teacher and instead permits an observational teaching style. This also potentiates the development and strengthening of essential skillsets (teamwork, leadership) beyond clinical and academic aptitude. 

Nonetheless, as mentioned above, the scarcity of research into the use of VR and AR technology within the context of anatomy teaching is a major limitation. As a form of computer technology, further advancements in development might necessitate newer applications and revised editions [40]. This is, however, an overall limitation to the use of technology in teaching and not a direct reflection on VR and AR alone.

As such, this systematic review aims to explore the literature available regarding the prevalence and use of VR/AR applications in undergraduate anatomy teaching. Additionally, this review will examine how these modalities are being integrated into the curricula, whether as primary instructional methods, alternatives to cadaver-based learning or as supplementary tools used alongside traditional anatomical dissection and prosection teaching, for the purpose of teaching medical students.

## Review

Methods

Selection Criteria

This report was concluded using the Preferred Reporting Items for Systematic Reviews and Meta-Analyses (PRISMA) guidelines. Using the search protocol, literature searches were conducted in four databases: MEDLINE, Scopus, Web of Science and ERIC. 

The following eligibility criteria, in Table 1 and Table 2 were set to select suitable literature.

**Table 1:** Inclusion criteria

Inclusion criteria
Published within the English language
Full text available within a peer-reviewed journal
Only presenting primary research
Published within 2009-2019
Population mentioned in the study were undergraduate medical/related students
Intervention was focused on visualisation technology specifically mentioning VR/AR technology
Intervention focused on anatomy

**Table 2:** Exclusion criteria

Exclusion criteria
Non-English texts
Literature published outside the date-range
Secondary research - reviews/editorials/books
Unpublished literature
Conference posters

Search Strategy 

A combination of free text and MeSH term searches was conducted to ensure sufficient scoping of the literature available.

The following search terms were designed: Group 1: referencing medical education; Group 2: referencing gross anatomy; and Group 3: referencing the intervention.

Table 3 illustrates the search terms designed.

**Table 3:** Search strategy

Search component	Filters applied
(undergraduate medical education OR learning OR teaching) AND (gross anatomy OR anatomy) AND (virtual reality OR VR OR augmented reality OR AR OR mixed reality)	English language; publication years (2009-2019)

Results

Data Extraction

**Figure 1:**
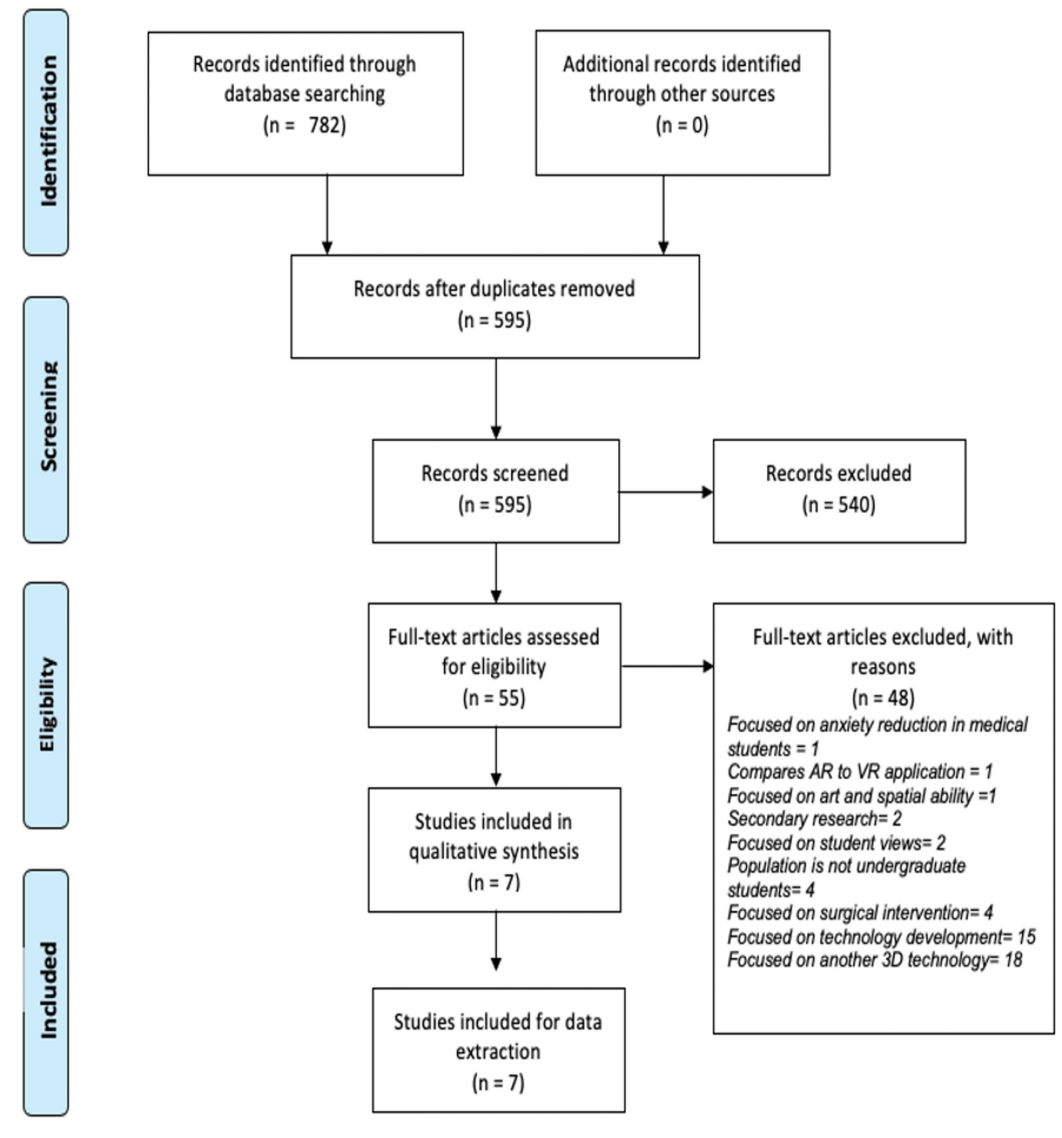
PRISMA chart

The literature searches performed produced a total of 782 papers, which were logged onto EndNote (Clarivate). Electronic removal of duplicate papers and manual cross-checking resulted in 595 papers that underwent primary and secondary data extraction text screenings, through full title and abstract screening. This process excluded 540 papers that were not compliant as per the inclusion and exclusion criteria. A final total of 55 papers were selected to undergo tertiary full-text screening, of which 48 papers were removed with reasons as illustrated in Figure 1. This resulted in seven conclusive papers, which were included for full qualitative synthesis in this report. 

Study Characteristics

The seven studies included for analysis were published between 2011 and 2019. All of the studies were randomised controlled trials (RCTs) and available in the English language. The studies were conducted in university anatomy laboratories across a range of different countries, including Germany, the United Kingdom, China and Turkey. The total study size (N) ranged from 16 to 120 participants, with an aggregated total of 427 participants across the seven studies. The median study population size was 66 participants. Only three out of seven studies accounted for age and gender distribution. The ages of the participants ranged from 18 to 35 years, in these studies, whilst the gender distribution was only narrowly dissimilar, with 130 males and 116 females in total. All three studies [32,34,38] reported no statistical significance attributable to the differences in baseline characteristics. 

The largest dissimilarity between the studies was the duration of exposure to the intervention, which had a range of 20 minutes to five hours. One paper, Deng et al. [32], did not explicate the exposure time and only stated that the control and interventional groups had equal in-study treatment. Two papers by Baratz et al. [29] and Küçük et al. [34] employed interventions based on AR technology (AR tablet application and AR mobile application), whilst the majority employed computer-based VR technology (VR forearm model, virtual human anatomy laboratory, VR Anatomage table).

A mixed-method approach, assessing quantitative and qualitative outcomes, was employed by all seven studies. Quantitative indicators were used to assess all the primary outcomes (academic competency); six studies employed subjective qualitative measures to assess their secondary outcome (student attitudes and perceptions). One study [34] qualitatively assessed both primary and secondary outcomes (academic competency and cognitive load). A complete outline of the study characteristics is available in Table 4.

**Table 4:** Methodology and key characteristics of included studies

Study, year	Study design	Participant size	Age	Gender	Aims	Intervention	Exposure to intervention
Aebersold et al., 2018 [33]	Randomised controlled trial	69 undergraduate nursing students; 34 - intervention, 35 - control	Not specified	Not specified	Assess changes in psychomotor skills	AR tablet application	20-25 minutes
Allen et al., 2016 [25]	Randomised controlled trial	47 undergraduate medical students; 22 - intervention, 25 - control	Not specified	Not specified	Examine educational efficiency of virtual 3D neuroanatomy model	Interactive VR learning resource based on virtual human project (VHP)	90 minutes
Baratz et al., 2019 [29]	Randomised controlled trial	16 undergraduate medical students; 8 - intervention, 8 - control	Not specified	Not specified	Assess qualitative experience of learning anatomy on Anatomage table vs dissection	Anatomage (VR) virtual dissection table	2 hours
Codd et al., 2011 [30]	Randomised controlled trial	39 undergraduate human anatomy students; 12- intervention modality, 14 - control, 13 - traditional modality	Not specified	Not specified	Determine if virtual reality (VR) teaching resources is as effective as traditional modalities used in basic anatomy teaching.	VR forearm application	30 minutes-1 hour
Deng et al., 2018 [32]	Randomised controlled trial	120 undergraduate medical students; 60 - intervention, 60 - control	Mean age 22.3 - intervention group, 22.5 - control group	Intervention: 31 males, 29 females; control: 32 males, 28 females	To assess the effect of digital virtual stimulation in gross anatomy teaching	Virtual human anatomy laboratory	Not specified
Küçük, et al., 2016 [34]	Randomised controlled trial	70 undergraduate medical students; 34 - intervention; 36 - control	Age ranges: 18-21 intervention, 18-21 control	Intervention: 18 males, 16 females; control: 16 males; 20 females	Determine effect of learning anatomy via mobile augmented reality (AR)	Mobile AR application	5 hours
Stepan et al., 2017 [38]	Randomised controlled trial	66 undergraduate medical students; 33 - intervention, 33 - control	Age ranges intervention: 21-25 (88%) 26-30 (9%) 31-35 (3%), control: 21-25 (91%) 26-30(9%) 30-35(0%), total: 21-25 (89%) 26-30 (9%) 31-35 (2%)	Intervention: 19 males, 14 females; control: 14 males, 19 females	To compare supplementary use of VR model to online textbooks	VR model	10 minutes

Primary Outcomes

All studies measured academic competency as their primary outcome. 

Multiple-choice and short-answer questions were used to assess academic competency. The interval between exposure to the intervention and assessment was narrow, with a range from five minutes to 20 minutes. Only one study [38] assessed extended knowledge retention, eight weeks post-interventional exposure, and found that there was no statistically significant difference (P = 0.47) in academic competency when using VR to teach gross anatomy, as opposed to cadaveric-based teaching, in long-term knowledge retention.

Only three [32-34] out of seven studies reported higher academic scores in the interventional groups, which were statistically significant (P = 0.0132, P = 0.01133 and P = 0.0534), indicating that MRT use within gross anatomical teaching can permit better academic results. 

The differences in the mean test scores attained by the interventional and control groups in these studies were 6.01% [32] (84.97 vs 78.96), 0.57% [33] (15.96 vs 15.39) and 9.8% [34] (78.14 vs 68.34). This illustrates that, although the students exposed to VR and AR technology demonstrated higher academic competency in the post-study tests, the differences in mean results ranged from distinctive to marginal.

All of the studies still utilised cadaver-based anatomy teaching as their primary teaching method. This was despite none of the studies concluding that this method of teaching was preferential in attaining higher academic test scores, in regard to statistical significance.

In addition to this, in all of the trials, the interventional study groups utilising AR/VR applications in anatomy scored higher on the post-study tests. Yet as mentioned, this was only significant in three studies.

All but one of the studies investigated the use of MRT as a supplement for cadaveric-based teaching and not as a substitution. This was reflected in the aims of the studies.

Furthermore, Allen et al. [25] found a statistically significant (P = 0.02), positive correlation between higher academic scores and increased spatial ability. In this same study, the interventional group utilising a ‘virtual human’ VR application scored higher in the post-study test. However, this was not significant as mentioned above.

Table 5 summarises the primary and secondary outcomes of each study included in this review.

**Table 5:** Outcomes and results of included studies

Study, year	Primary outcome	Secondary outcome	Results	Conclusions	Limitations
Aebersold et al., 2018 [33]	Academic competency of intervention	Student attitudes and perceptions towards using intervention	Statistically significant difference in academic performance in favour of the intervention group: intervention, 15.96 ± 0.75; control, 15.39 ± 1.01 (P = 0.011).	AR is effective for procedural skills training.	Small study population single-institute study. Students had prior exposure to measured variable.
Allen et al., 2016 [25]	Academic competency of intervention	Association between academic score and spatial ability; student attitudes and perceptions towards using intervention	Overall (Pre-quiz, Quiz 1 and Quiz 2), no statistically significant difference in academic performance between the intervention and control groups. Statistically significant correlation between academic score and spatial ability (r = 0.22, P = 0.04; r = 0.25, P = 0.02; r = 0.26, P = 0.02). Students reported ‘greater ease with visualisation using the 3D tool’ (mean = 4.24 ± 0.92; range, 1-5; P = 0.05). Incorporating the 3D model enabled better understanding of neuroanatomy compared with traditional learning resources (mean = 4.29 ± 0.79).	3D learning resources improve student anatomical knowledge in regard to spatial ability.	Small study population. Time spent utilising intervention was not standardised across the groups.
Baratz et al., 2019 [29]	Academic competency of intervention	Student attitudes and perceptions towards using intervention	No statistically significant difference between the intervention and control groups in mean examination scores. Pelvis and peritoneum examination: Intervention, 69.9% ± 6.8%; Control, 69.1% ± 7.4% (P = 1.00). Musculoskeletal examination: Intervention, 74.9% ± 6.5%; Control, 70.8% ± 10.6% (P = 0.41). Statistically significant student reflections on the intervention. Greater amount learned: 3.68 ± 0.94 vs 2.99 ± 1.06 (P = 0.01). Greater degree of excitement for sessions: 3.51 ± 0.96 vs 2.30 ± 1.09 (P = 0.01).	Anatomage table reported greater student excitement and increased perception of knowledge attainment.	Small study population limited student access to Anatomage table selection bias: volunteers actively pursued opportunity to learn anatomy using technology.
Codd et al., 2011 [30]	Academic competency of intervention	Student attitudes and perceptions towards using intervention	No statistically significant difference between the intervention and traditional modality (P = 0.5). Statistically significant differences were observed between the intervention/traditional groups and the control group, with the intervention and traditional groups reporting higher scores (P < 0.001). Student feedback surveys indicated that the VR application was not as effective as dissection or prosection but was superior to standalone textbook use.	VR application meet initial aims and thus was incorporated into the module as a supplementary resource.	Small study population traditional group had an increased learning time final assessment did not occur at the same time.
Deng et al., 2018 [32]	Academic competency of intervention	Student attitudes and perceptions towards using intervention	Statistically significant difference in academic performance in favour of the intervention: intervention, 84.97 ± 7.86; control, 78.96 ± 5.78 (P < 0.001); 100% of students in the intervention group reported satisfaction with the teaching reform.	Digital virtual stimulation in gross anatomy enhances theoretical and practical learning. Supplementing traditional modalities with DVS is superior to conventional means alone.	Single institute study
Küçük et al., 2016 [34]	Academic competency of intervention	Student attitudes and perceptions towards using intervention; cognitive load	Statistically significant difference in academic performance in favour of the intervention: intervention, 78.14 ± 16.19; control, 68.34 ± 12.83 (P < 0.05). Statistically significant difference in cognitive load with the intervention: intervention, 3.88 ± 1.71; control, 4.86 ± 1.85 (P = 0.05).	Mobile AR applications can report higher academic achievement. But application should be further investigated.	Cognitive load is ductile and changes with environment Student also spent independent time assessing materials.
Stepan et al., 2017 [38]	Academic competency of intervention	Student attitudes and perceptions towards using intervention	No statistically significant difference in academic performance with the intervention. Pre-intervention quiz: 0.61 ± 0.21 vs. 0.60 ± 0.20 (P = 0.86). Post-intervention quiz: 0.76 ± 0.14 vs. 0.75 ± 0.16 (P = 0.87). Retention quiz: 0.68 ± 0.25 vs. 0.64 ± 0.23 (P = 0.47). Statistically significant student reflections on the intervention: more engaging (72.45 ± 19.68 vs. 15.67 ± 14.19, P < 0.01), more enjoyable (82.52 ± 17.07 vs. 17.55 ± 14.47, P < 0.01), and more useful for learning (63.76 ± 17.97 vs. 48.88 ± 23.57, P < 0.01).	VR model is as academically effective as academic traditional teaching. Increased quality and accessibility will make VR more accessible.	Short learning time; short exposure to intervention.

Discussion

Aims

This review aimed to evaluate the prevalence of AR and VR use in gross anatomy teaching, the capacity (primary or supplementary) in which the technology was used, and the factors influencing the use of this technology in anatomy teaching. 

Application of MRT in Gross Anatomical Teaching 

The collective results of the primary outcomes from the included studies do not support the usage of MRT applications as a substitute for cadaver-based anatomy teaching. 

Out of seven studies, one [32] reported within its conclusion that the use of an AR mobile application can attain higher academic scores than cadaver-based teaching; however, this was conjoined with recommendations to seek further research into its application. As mentioned, despite three studies [32-34] reporting statistically significant, higher academic scores, none of the studies explicitly recommended or implied the use of MRTs to teach anatomy. Deng et al. [32] stated that using MRTs to supplement cadaver-based anatomy teaching was ‘more superior’ than employing traditional means alone, despite higher test scores being attributed to the interventional group utilising the VR application.

A plausible conclusion from this is that further compound research is needed to fully compare this emerging technology with the conventional ‘gold standard’ [40,41], cadaver-based teaching.

This was also mentioned by two studies [34,38], which incentivised the need for further research to assess the long-term implications of primarily using MRTs to teach anatomy. 

Codd et al.’s study [30] implemented the use of a VR application as a supplementary learning modality to cadaver-based anatomy; this study, however, reported no statistically significant difference in the use of VR. Yet in this study, the use of the VR application and cadaveric dissection was found to be better than the control (anatomical textbooks), suggesting that, despite VR not showing greater academic potential, it could be a more effective supplementary learning modality. 

The majority of the studies (five out of seven papers) investigated the use of VR applications, in preference to AR applications. The precise cause of this was not mentioned in either study; however, current research has proposed greater potential for the use of VR as opposed to AR [42]. This could be derived from the fact that VR technology permits a greater depth of immersion into the virtual world due to its inherent design (HMD) [43]. Accompanied by sophisticated computer software, this could provide a highly tailored digital construct, which is fitting for studying and investigating anatomical structure which encompass great detail and precision by design [44].

From VR applications in the gaming industry [45], it is known that this technology can be extensively manipulated, creating very realistic computer graphics and animations [45]. This might add some context to the preferential use of VR as opposed to AR.

However, the majority of applications use VR despite the positive attributes associated with AR technology. In contrast to VR, AR is very widely accessible, as previously mentioned. It is also much further adapted for use in medical education [46] as it has a current market as a supplementary medical anatomy tool in the context of mobile apps [47].

Perhaps, investigative research should be conducted extensively, contrasting the two different mixed reality technologies, alongside further research into their competence in anatomical teaching.

There is currently no research into the long-term efficacy of MRT use in anatomy teaching of undergraduate medical students. This makes it difficult to conclude any drastic changes to anatomy curricula. However, there are also institutions that solely employ the use of MRTs to teach anatomy [48], yet the rationale behind this is unclear, as the supportive research and evidence are not presently available. 

Influencing Factors 

Positive student perception and satisfaction with VR/AR interventions were reported in all but one [30] of the studies. This reflected current literature, which reports that the use of technology in education is very attractive to students [48-53] in spite of efficacy [50]. 

Within the conclusion, five [25,29,32,34,38] out of seven studies focused on and emphasised the reported student perceptions and attitudes towards the use of VR/AR in anatomy teaching. Although this was not a primary outcome in any study, it is still a substantial outcome to consider.

As previously stated, student perceptions of their learning directly influence motivation [54], which is a dependent factor to academic performance [55].

As the main beneficiaries of adaptations in medical teaching, students should be involved in the investigative stage of evaluating new teaching methods and modalities. However, as research has shown [56], student perceptions are not largely reflective of actuality. This was reflected by one study [29] which reported that students perceived that they ‘learned a greater amount of information’ [29] with the use of the VR Anatomage table. This reflection was found to be statistically significant, but the academic scores attained showed that there was no statistical significance in the difference in academic test scores. The differences in test scores in the two assessments recorded for the intervention and control group were also not drastic (PPS exam: intervention, 69.9% ± 6.8%/control, 69.1% ± 7.4%, P = 1.00; MSK exam, intervention: 74.9% ± 6.5%/control: 70.8% ± 10.6%, P = 0.41, with calculated differences of 1.14% 5.47%, respectively.

Moreover, there was also potential bias from the participants, which could have skewed this result. All of the participants in the trials reviewed were volunteers who were openly recruited. This could have attracted a large percentage of participants who were already intrigued by visualisation technology or exposed to it. Similarly, only one study [38] assessed prior exposure to visualisation technology outside of the study (video games, graphics software) as part of baseline characteristics assessment, further indicating potential bias. None of the studies, which excluded this, addressed it within their discussions.

It’s also worth reflecting on the fact that technology is a standard feature of education today [51] and undergraduate medical schools are not excluded from this exposure. A broad shift towards CBL [48,52] and supplementary teaching via mobile technology could arguably be another influential factor in the use of MRTs in teaching gross anatomy to medical students. 

There are numerous mobile applications and 3D computer software on the market and in use, which supplement cadaver-based teaching [53]. These applications are employed both by institutions at large and individually by students [54-60]. Hence, there is a clear market and demand for the use of MRT in anatomy teaching. 

Limitations

The exclusion of newer studies and grey literature is a major limitation of this review. The prevailing research could have been confined within grey literature, and hence, this exclusion limited the breadth of this review and introduced publication bias. The rationale for this exclusion was the difficulty of constructing a vigorous grey literature search strategy and eligibility criteria.

A secondary limitation is the exclusion of non-English literature, which again limited the scope of studies available for review and allowed for language bias. Furthermore, the inclusion of a full meta-analysis, which is a quantitative statistical analysis, accompanying this review would have strengthened the overall power of this review. This would have added to the reliability of the review.

Future Directives

This review has signified the need for further research to be conducted within this area of medical education. Larger studies completed over greater periods of time should be conducted, with a strong focus on efficacy, long-term knowledge retention and clinical transferability, as there is currently no research in these areas. Furthermore, additional associated factors, which may impact the potential implications of VR and AR applications in undergraduate anatomy, should also be investigated and evaluated. These include the potential cost, long-term maintenance and training of staff who are skilled to manage medical technology.

## Conclusions

Current research is not sufficient to substantiate a conclusion on MRT use in undergraduate medical gross anatomy teaching. However, there is agreement amongst the studies included in this review that mixed reality technologies have a strong potential in anatomy teaching as supplementary teaching modalities. VR applications are more considered than AR applications with reference to this review, as potential learning modalities in undergraduate medical anatomy teaching. The centuries-old practise of cadaver-based anatomy teaching is, however, still as relevant and efficient today as it was at its inception.
